# Transcatheter heart valve in valve implantation with Edwards SAPIEN bioprosthetic valve for different degenerated bioprosthetic valve positions (First Iranian ViV report with mid-term follow up)

**DOI:** 10.15171/jcvtr.2017.26

**Published:** 2017-09-30

**Authors:** Ali Mohammad Haji Zeinali, Kyomars Abbasi, Mohammad Saheb Jam, Shahrooz Yazdani, Seyedeh Hamideh Mortazavi

**Affiliations:** Tehran Heart Center, Tehran University of Medical Sciences, Tehran, Iran

**Keywords:** Bioprosthetic Valve, Transcatheter Valve, Valve-in-Valve, Edwards SAPEIN Valve

## Abstract

***Introduction:*** After early successful experience with transcatheter aortic valve replacement (TAVR), concept of transcatheter implantation of a new valve within a failing bioprosthetic valve emerged. Valve-in-valve (ViV) implantation seems to be a simpler option for high risk surgical patients.

***Methods:*** We performed five ViV procedures in different valve positions. We included patients with failing bioprosthetic valves with high surgical risk due to concomitant comorbidities. We performed 2 transapical ViV procedures for failing mitral bioprosthetic valves, 1 transfemoral procedure for failing pulmonary valve and 2 transfemoral ViV implantation for failing tricuspid bioprosthetic valves.

***Results:*** The procedures were successfully completed in all 5 cases with initial excellent fluoroscopic and echocardiographic verification. There was no valve embolization or paravalvular leakage in any of the cases. Transcatheter valve function was appropriate with echocardiography. Post procedural clinical adverse events like pleural effusion and transient ischemic attack were managed successfully. In midterm follow up all cases remained in appropriate functional class except from the transcatheter pulmonary valve which became moderately stenotic and regurgitant.

***Conclusion:*** As the first Iranian all-comers case series with midterm follow up for ViV implantation, we had no mortality. Interestingly none of our patients had neurologic sequelae after the procedure. Midterm follow up for our patients was acceptable with good functional class and appropriate echocardiographic findings. Due to high surgical risk of the redo procedure after failing of a bioprosthetic valve especially in elderly patients with comorbidities, ViV implantation would be a good alternative to surgery for this high risk group.

## Introduction

The first human transcatheter aortic valve implantation (TAVI) was reported in a patient with severe aortic stenosis (AS) presenting with cardiogenic shock in 2002.^1^ During recent years great progress was achieved in TAVI for native degenerated aortic valves.^2^ Different transcatheter heart valves were designed^3^ and long term follow up are available.^4^ Apart from their well-known indication, available transcatheter valves are finding another attractive indication in treating patients with failing bioprosthetic valves in other valve positions. Due to favorable clinical results, bioprosthetic valves have increasingly been chosen over mechanical valves even in younger patients undergoing surgical valve replacements. Many patients with previous surgical bioprosthetic valve replacement had valve degeneration during time.^5^ Surgical Redo procedure for failing bioprosthetic valves is challenging in some patients due to high surgical risk. Operative mortality ranges from 1.5% to 23% depending on the clinical scenario.^6^

The concept of transcatheter implantation of a new valve within the failing bioprosthetic valve (ViV) seems to be a simpler option and initiated since 2007.^7^

Indications for ViV implantation include bioprosthetic stenosis, regurgitation, or both.‏

Case reports from Europe and Canada confirmed successful implantation of off-label TAVI valves within failing bioprosthetic valves.^8, 9^

To contribute this growing knowledge and manage the high risk patients who had not any surgical chance, we made ViV implantation in different valve positions and now report our first all comers cases with mid-term follow up as the first report from Iran.

## Materials and Methods

From February 2015 to October 2015, five patients were admitted to our institution with significant signs and symptoms of bioprosthetic valve dysfunction (Table 1). Indication for valve replacement was defined as the current guidelines. Those with severe commodities which exclude them from surgery based on the decision of the interdisciplinary heart team, were entered the study. Coronary angiography was performed to rule out significant coronary artery disease requiring intervention. Transthoracic and transesophageal echocardiography were done to ascertain that other valves and ejection fraction were acceptable and not responsible for symptoms.

**Table 1:** Demographic and characteristics of the patients

**Patient number**	**Age**	**Sex**	**Diseased valve**	**Bioprosthetic valve**	**Time of surgery**	**Concomitant Cardiac disease**	**EF (%)**	**CAD**	**PAP ** **(mm Hg)**
1	77	F	MS	St Jude 31 mm	11 y ago	None	55	No	29
2	76	F	MS&MR	St Jude 31 mm	10 y ago	Previous CABG + Permanent pacemaker	45-50	mild	50-55
3	29	M	PI&PS	Medtronic 25	7 y ago	Previous tetralogy of fallot correction	50	No	20
4	59	F	TS&TR	Mosaic 31	1 y ago	MVR	50	No	30
5	55	F	TS&TR	Mosaic 31	4 y ago	MVR & AVR	45	No	25

Abbreviations‏: MS; mitral stenosis, MR; mitral regurgitation, TS; tricuspid stenosis, TR; tricuspid regurgitation, PS; pulmonary stenosis, PI; pulmonary insufficiency, EF; ejection fraction, CAD; coronary artery disease, PAP; pulmonary artery pressur

Two cases required valve implantation in bioprosthetic mitral position. In one case bioprosthetic pulmonary valve had involved and 2 cases had bioprosthetic tricuspid valve malfunction. Table 1 and Table 2 summarize important demographic, clinical and technical data regarding cases. All procedures were performed in a hybrid operation room with general anesthesia but from different access sites. Routine lab exams and premedications for an open heart surgery were done for all patients.

**Table 2:** Procedural characteristics of the patients

**Patient number**	**Diseased valve**	**Approach**	**New Valve (Edwards SAPIEN, mm)**	**PG before procedure (mm Hg)**	**PG after procedure (mm Hg)**	**Paravalvular leakage**	**Complication (procedural and follow up)**	**Pacing chamber**
1	MS	Transapical	29	24	4	No	left PE and TIA	RV
2	MS&MR	Transapical	29	28	9	No	Left PE	RV
3	PI&PS	Transfemoral vein	23	59	10	No	Mild valve restenosis	RV
4	TS&TR	Transfemoral vein	26	16	5	No	None	None
5	TS&TR	Transfemoral vein	29	15	4	No	Warfarin toxicity	LV

Abbreviations‏: MS; mitral stenosis, MR; mitral regurgitation, TS; tricuspid stenosis, TR; tricuspid regurgitation, PS; pulmonary stenosis, PI; pulmonary insufficiency, PG; peak gradient, PE; pleural effusion, RV; right ventricle, LV; left ventricle

### Case 1

**Figure 1:**
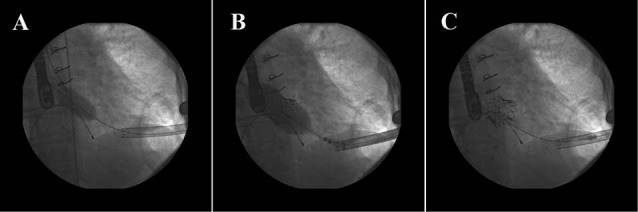
Transapical Edwards SAPIEN valve in valve implantation in degenerative Mitral Bioprosthetic position. A. Edwards Ballooon predilation of degenerative Mitral valve from Edwards transapical sheath under TEE control and RV rapid pacing. B. Edwards SAPIEN valve 29 mm implantation. C. Final position of new Edwards SAPIEN valve in previous Mitral valve in AP fleuroscopic view.

A 77-year old lady with history of mitral valve replacement (MVR) 11 years ago with St Jude bioprosthetic valve 31 mm had symptomatic severe stenosis. In hybrid operation room under general anesthesia her cardiac apex was explored by intercostal apical incision and the Edwards apical sheath 20 Fr was inserted in left ventricle (LV). Temporary pacemaker lead for rapid ventricular pacing was inserted in right ventricle (RV). A 0.035 inch standard guide wire was passed through the LV sheath via the bioprosthetic mitral valve into the left atrium (LA). Balloon predilation for bioprosthetic mitral valve was done with Edwards balloon 24 mm. Finally transapical Edwards SAPIEN XT (Edwards Lifesciences, USA) bioprosthetic valve 29 mm was implanted over the previous prosthetic mitral valve ring that was well marked on fluoroscopy and simultaneous echocardiography under rapid ventricular pacing. Final LV injection and control transesophegeal echocardiography (TEE) revealed appropriate position of the new valve with 4mm Hg pressure gradient without any mitral regurgitation (MR) and paravalvular leakage (Figure 1). The day after procedure, the patient had in hospital symptoms of mild agitation and disorientation. Brain imaging ruled out any neurologic deficit. Three weeks after discharge she was readmitted due to cough and dyspnea. Work up revealed significant left sided pleural effusion which was managed medically. Six month clinical and echocardiographic follow up revealed functional class I to II with acceptable Edwards bioprosthetic mitral valve function with 4 mm Hg pressure gradient.

### Case 2

**Figure 2:**
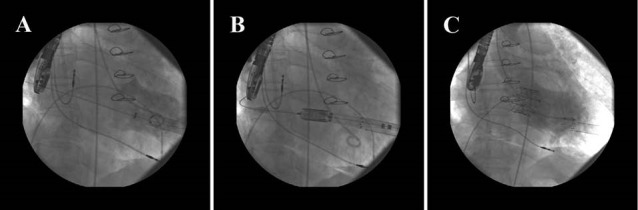
Transapical Edwards SAPIEN valve in vale implantation in mitral position. A. LV angiography after transapical sheath insertion under TEE. B. Edwards SAPIEN valve 29 mm before implantation in mitral ring position. C. Final LV graph revealed good position of new valve.

A 76-year old lady with St Jude bioprosthetic valve 31 mm in mitral position, that was replaced 10 years ago concomitant with coronary artery bypass grafting, was degenerated leading to concomitant mitral stenosis (MS) and MR. The patients had permanent pacemaker, too. In hybrid operation room under general anesthesia and minimal subcostal LV apex exploration an Edwards apical sheath 20 French was inserted into LV. Temporary pacemaker for rapid ventricular pacing was inserted into the right ventricle (RV). A standard 0.035 inch guide wire was passed through the LV via the bioprosthetic mitral valve into the LA. Bioprosthetic mitral valve predilation with an Edwards Balloon 24 mm was done and then a transapical Edwards SAPIEN valve 29 mm was implanted in mitral position. Final LV injection revealed no MR and control TEE showed appropriate valve position with no paravalvular leakage (Figure 2). In early follow up patient re admitted with shortness of breath which was due to severe left sided pleural effusion that successful drainage and management was done. Six month clinical and echocardiographic follow up revealed functional class I and mean mitral valve pressure gradient 9 mm Hg.

### Case 3

**Figure 3:**
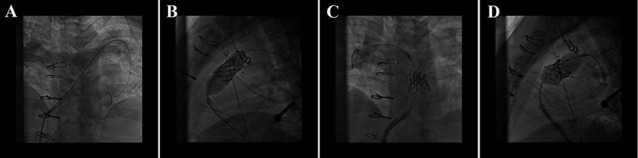
Transfemoral vein approach for Edwards SAPIEN valve in valve implantation in previous Bioprosthetic Pulmonary valve position in a tetralogy of fallot patient. A. Passing the stiff wire from right femoral vein to Pulmonary artery in AP view. The three markers of previous valve were seen. B. Balloon Expandable stenting of previous valve in lateral view. C. Positioning of new Edwards SAPIEN valve in mid of previous stent in AP view. D. Implantation of Edwards SAPIEN valve 23 mm in pulmonary position in lateral view.

A 29-year old man who had surgical correction of tetralogy of fallot (TOF) in childhood near 25 years ago and underwent pulmonary valve replacement (PVR) 7 years ago with Medtronic valve 25 mm, recently developed prosthetic pulmonary valve stenosis. Under general anesthesia a long sheath 16 Fr was inserted into the right femoral vein and after right heart catheterization a super stiff 0.035 inch guide wire was put in pulmonary artery. For predilating the stenotic pulmonary valve, Tyshak balloon size 25 mm was used. Then RV out flow tract (RVOT) stenting with CP stent 34 mm was done over the Landerquest super stiff wire. Finally bioprosthetic transfemoral Edwards SAPIEN valve 23 mm was implanted in the stented RVOT. Final right heart catheterization and PA injection revealed trivial (most probably catheter induced) pulmonary insufficiency (PI) and less than 10 mm Hg gradient across the new prosthetic valve (Figure 3). The patient discharged without any complication. Six month clinical follow up revealed near normal functional class but echocardiography revealed bioprosthetic PV with increased gradient (MG: 36 mm Hg) with moderate pulmonary insufficiency.

### Case 4

**Figure 4:**
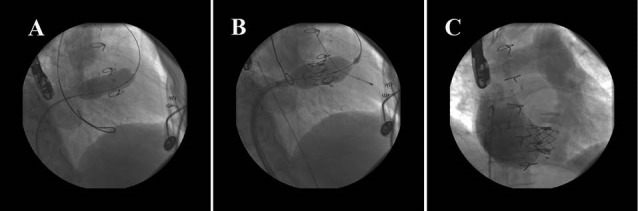
Transfemoral vein Edwards SAPIEN valve in valve implantation in degenerative Tricuspid valve. A. Edwards Balloon predilation of degenerative Tricuspid valve. B. Edwards SAPEIN valve 26 mm implantation under LV rapid pacing. C. RA graph revealed good position of the new valve without TR.

A 59-year old lady had degeneration of previous tricuspid bioprosthetic MOSAIC valve 31 mm (Medtronic, USA) which had been replaced one year ago with concomitant MVR, and now had symptomatic TS and TR. Due to high surgical risk she was planned to undergo interventional ViV implantation in the tricuspid position. In hybrid operation room under general anesthesia an 18 French sheath was inserted percutaneously in right femoral vein. A temporary pacemaker lead was inserted in LV through left femoral artery sheath for rapid ventricular pacing. Via right femoral vein sheath a 0.035 inch Landequest super stiff wire was placed in distal pulmonary artery bed. Predilation of degenerated bioprosthetic tricuspid valve was done with Edwards balloon 23 mm. Consequently transfemoral Edwards SAPIEN bioprosthetic valve 26 mm was implanted in the tricuspid position over the previous mosaic valve ring. Control TEE and RA injection revealed appropriate position of the valve (Figure 4). The pre procedure tricuspid regurgitation was disappeared and the tricuspid valve peak gradient decreased from 16 mmHg to 5 mmHg. Six month clinical and echocardiographic follow up revealed significant improvement of symptoms and good function of the new valve with acceptable gradient.

### Case 5

**Figure 5:**
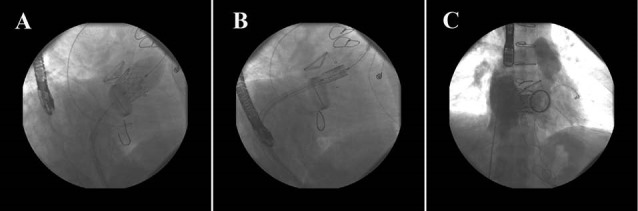
Transfemoral vein Edwards SAPIEN valve in valve implantation in degenerative Tricuspid valve position. A. Edwards SAPIEN valve in Tricuspid valve position. Three markers of previous Tricuspid valve were seen with prosthetic mitral and aortic valves. B. Implantation of new valve under TEE without any pacing. C. RA graph revealed good position of new tricuspid valve and other previous prosthetic mitral and aortic valves and epicardial pacemaker lead.

A 55-year old lady had symptomatic degeneration of previous MOSAIC (Medtronic, USA) tricuspid bioprosthetic valve 31 mm which was replaced 4 years ago concomitant with MVR and AVR. In hybrid operation room under general anesthesia a 22 French sheath was inserted into the right femoral vein. A Landequest super stiff wire was placed in distal pulmonary artery bed. Predilation of degenerated bioprosthetic tricuspid valve was done with Edwards balloon 25 mm. Consequently transfemoral Edwards SAPIEN bioprosthetic valve 29 mm was implanted in the tricuspid position. Control TEE and RA injection revealed appropriate position of the valve without any TR and 4 mm Hg pressure gradients across the valve (Figure 5). About 3 weeks after new valve implantation patient was re admitted due to warfarin toxicity with INR 8.8 which was managed uneventfully. Six month clinical and echocardiographic follow up revealed functional class I with acceptable new valve function similar to the predischarge indices. Tricuspid mean gradient after 6 month was 6 mmHg with no TR and paravalvular leakage.

## Results

The procedures were successfully completed in all 5 cases with initial excellent fluoroscopic and echocardiographic verification. There was no valve embolization or paravalvular leakage in any of the cases. Transcatheter valve function was appropriate with echocardiography. Post procedural clinical adverse events like pleural effusion and transient ischemic attack were managed successfully. In midterm follow up all cases remained in appropriate functional class except from the transcatheter pulmonary valve which became moderately stenotic and regurgitant.

## Discussion

Since transcatheter heart valve (THV) is an invaluable and minimally invasive procedure, it could be considered as an alternative method for open redo surgical valve replacement in high-risk elderly patients with bioprosthetic valve dysfunction. Valve-in-valve (ViV) terminology has been originated for THV implantation in a degenerated bioprosthesis.^7^

Wenaweser et al has reported the result of the first in man aortic valve ViV procedure with a CoreValve Revalving System in an 80-year patient who had a failed Mitroflow bioprosthesis. They showed that the procedure was easy to do and associated with immediate improved hemodynamic status.^10^

In 2007 the ViV concept in mitral position was initially tested in 7 pigs with success in all cases.^7^

Two years later in 2009, the first transapical transcatheter mitral ViV implantation in a human was done by Cheung and colleagues. A cuffed, 26-mm Cribier-Edwards transcatheter valve (Edwards Lifesciences LLC, Irvine, CA) was deployed within the mitral xenograft. After the operation, the transcatheter valve function was proper however due to multiple organ dysfunction, the patient died.^11^

In 2010 Hon et al reported the first transatrial transcatheter tricuspid ViV implantation in a human. They successfully deployed a 26-mm Edwards SAPIEN balloon expandable bioprosthesis (Edwards Lifesciences, Irvine, CA) into a severely stenotic tricuspid bioprosthesis.^12^

In the same year (2010) the first total percutaneous (transfemoral) tricuspid ViV was performed successfully.^13^

A large Canadian multicenter experience with ViV series described‏ 24 patients with failed aortic, mitral,‏ pulmonary, and tricuspid bioprostheses. In this study, different approaches were used for implantation of Edwards transcatheter valves: in aortic and mitral bioprostheses, transapical or transfemoral approach was used. In pulmonary valves, transvenous approach was administered and direct right atrial access was used for the tricuspid valve. They reported an‏ overall procedural success rate of 96% and a 30-day mortality‏ of 4.2% which shows an early learning curve. One of their patients (4%) developed stroke. None of the patients required pacemaker. Considering the bioprosthetic position, we found 0% 30-day mortality for aortic, pulmonary and tricuspid valves and 14% for mitral valve.^14^

Conradi and colleagues performed 75 ViV procedures from 2008 to 2014. THVs used were Edwards SAPIEN (XT)/SAPIEN, Medtronic Core Valve/Core Valve Evolut(R), St Jude Portico, Boston Scientific Lotus, Jena Valve, and Medtronic Engager. Overall immediate procedural (≤72 hours) and all-cause 30-day mortality were 2.7% and 8.0%. No periprocedural strokes or cases of coronary obstruction occurred. There were less than or equal to mild paravalvular leakage in all of the study cases. Based on their finding, they concluded that ViV can be conducted in high risk patients in all anatomic positions which is associated with favorable hemodynamic and clinical outcomes.^15^

Our early experience with ViV implantation is very promising. Our procedural success rate in the mentioned 5 cases was 100%. We had no major neurologic deficit after the procedures. Only one patient suffered from a mild transient ischemic attack (TIA) with no persistent deficit. After discharge 3 patients readmitted, two for pleural effusion and one for warfarin toxicity with eventual discharge. Midterm follow up with echocardiography 3 to 6 months after the procedures revealed appropriate valve position with acceptable function. These early results suggest that ViV procedure is completely feasible in high surgical risk patients. We utilized Edwards SAPIEN transcatheter valve for ViV implantation in mitral, tricuspid and pulmonary positions. CoreValve was not implanted in our patients due to its greater length with excessive protrusion into adjacent cardiac chambers with potential valve interferences. In available case reports CoreValve has been implanted for ViV procedure in aortic position successfully. Data about newer transcatheter valves including Portico, Directflow and other recent innovative valves are scarce. With introduction of these new transcatheter valves, ViV procedures would progress greatly in the near future.

There are few studies reporting the results of ViV implantation in whom only SAPIEN has been implanted. Regarding the ViV implantation in mitral bioprostheses, a multicenter Canadian study has reported 2 cases of mortality. The first in-man experience through the percutaneous transseptal approach was unsuccessful due to the valve embolization in to the left ventricle, as well as the emergent surgical conversion and death.^14^

There are different approaches to access the mitral valve such as transseptal and transatrial. Nevertheless, most experience has been gained using the transapical access.^11^

We performed 2 cases in mitral position and our approach was transapical. These 2 patients are event free during 6 month follow up but both of them readmitted shortly after discharge due to massive left sided pleural effusion and our only TIA occurred in one of these patients.

In the tricuspid position, ViV implantation has been performed in few patients. Right internal jugular vein or transfemoral venous approach has been used in successful tricuspid transatrial implantation before. We performed 2 cases in this valve position with excellent results.

Implantation of permanent pacemaker has shown to be less frequent in ViV procedure than TAVI.^16^ None of our patients required permanent pace maker after the procedure.

Four percutaneous devices have been yet introduced for implantation of transcatheter valve in failed bioprosthetic valve cases including the Edwards SAPIEN and its relations, the Medtronic CoreValve, the Medtronic Melody valve and St Jude Portico valve. The SAPIEN could be used in all four valve positions because of its short delivery system and cylindrical structure. The Melody valve is used for pulmonic and mitral valves and the CoreValve is only used for the aortic position.

As mentioned earlier, ViV implantation could be considered as an alternative method for treatment of degenerated bioprostheses in high risk elderly patients. This technique has been previously confirmed technically through in vitro and in vivo studies.^17^

There are few case series for mitral ViV with the Melody or Edwards SAPEIN valves. Because valve’s length might interfere with aortic outflow tract as well as the mitral subvalvular apparatus, there is no corevalve implantation so far.^11,18^

Both the Edwards SAPIEN and the Melody valves have been reported for tricuspid ViV procedures.^19^

Similar to the original TAVI cohorts, patients for consideration of the ViV procedure should undergo a multidisciplinary team evaluation involving cardiologists, cardiothoracic surgeons, anaesthetists, nurses and frequently geriatricians.

In our center after obtaining good and widespread experience in endovascular treatment of complex cardiovascular and structural heart diseases and TAVR, we started our program for ViV.^20,21^ For TAVR according to situation we used to perform the procedure both transfemorrally and transapically. Our heart team progressed a lot during recent years both in obtaining access and the whole procedure for TAVR. This experience with TAVR became our cornerstone for initiating the ViV program. With defining ViV program within 5 months we performed five ViV cases successfully.

Due to increasing numbers of patients with failing bioprosthetic valves we expect more cases suitable for ViV in near future. To sum up according to the literature and also our early experience, ViV is completely feasible and safe for treatment of failing bioprosthetic valves especially in patients with high surgical risk. In future reports we will declare our long term follow up to delineate the efficacy of ViV.

## Conclusion

Transcatheter ViV implantation for treatment of high risk patients with previous degenerated bioprosthetic valves is a new technique which proved to be safe in well experienced structural heart teams. Midterm follow up results are satisfactory. Edwards SAPIEN bioprosthetic valve can be used in all four heart valve positions for ViV implantation.

## Ethical Approval

The present study was approved by the Institutional Review Board and Ethics Committee of Tehran Heart Center.

## Competing interests

The authors declare there is no conflict of interest.
